# A simple *in vitro* method to evaluate the toxicity of functional additives used in shrimp aquaculture

**DOI:** 10.1016/j.mex.2018.01.010

**Published:** 2018-01-31

**Authors:** Cristóbal Domínguez-Borbor, Bolívar Chalén-Alvarado, Jenny A. Rodríguez

**Affiliations:** ESPOL Polytechnic University, Escuela Superior Politécnica del Litoral, ESPOL, Centro Nacional de Investigaciones Marinas (CENAIM), Campus Gustavo Galindo Km. 30.5 Vía Perimetral, P.O. Box 09-01-5863, Guayaquil, Ecuador

**Keywords:** Viable hemocyte, Tetrazolium salts, Metabolism

## Abstract

To mitigate the economic losses provoked by disease outbreaks, shrimp producers employ therapeutic additives. However, important issues such as the toxicity of these products on shrimp are not always considered. *In vivo* toxicity assays require a lot of time and large economic and physical resources. Here, we describe an *in vitro* procedure to evaluate the toxicity of functional additives, used in the production of shrimp *Penaeus vannamei*. This method adapted the cell viability assay based on the reduction of tetrazolium salts (MTT) to primary cell cultures of shrimp hemocytes.

• A simple and reliable tool that requires few physical and economic resources to evaluate in short time (6 h) the cytotoxic effect of therapeutic products and additives to be included in shrimp culture
• This inexpensive method requires only a modified Hank's balanced salt solution (HBSS) containing Ca^2+^ and Mg^2+^ to keep hemocytes metabolically active to successfully carry out the cytotoxicity assay
• This toxicity *in vitro* assay does not require exposure of the shrimp to compounds at toxic concentrations.

## Graphical abstract

**Figure:**
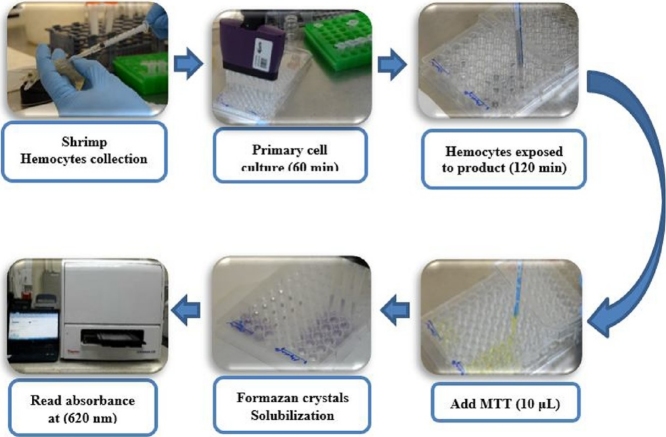

## Method details

### Background

The expansive growth of the shrimp aquaculture industry is accompanied by the disease outbreaks. To mitigate the economic losses, shrimp producers employ therapeutic additives, such as antibiotics, immune modulators, organic acids, essential oil and antioxidants. However, important issues such as the toxicity of these products on shrimp are not always considered. While *in vivo* toxicity assays require considerable time and economic resources [1], the development of easy and robust *in vitro* protocols is highly relevant. The cell viability assay developed by Mosman [2], based on the reduction of tetrazolium salts (3-(4,5-dimethylthiazol-2-yl) −2,5-diphenyltetrazolium bromide) (MTT), is widely used to measure *in vitro* cytotoxic in eukaryotic cells [[2], [3]]. To develop an *in vitro* toxicity test suited for the assessment of the toxicity of feed additives for shrimps, we adapted this Mosman protocol to primary cell cultures of shrimp hemocytes. This fast and inexpensive assay can be used by the shrimp industry to determine non-toxic therapeutic doses of functional additives as a pre-application process in *in vivo* trials, and shrimp farms.

### Materials

#### Reagents

Acid chloride (1 N)

Ethanol (70% [v/v] in distilled water)

Calcium chloride solution (Ca Cl_2_) (1 M; filtrated on a 0.22 μm filter)

Citric acid solution 1 N

Formaldehyde (4% [w/v] in distilled water)

Hanks balanced salt solution (HBSS 10x) (Gibco 14185-052)

Hepes solution (kept at 4 °C) (1 M; filtrated on a 0.22 μm filter)

Hydrochloric acid solution 1 N

Isopropanol (kept at 4 °C)

Magnesium chloride solution (Cl_2_Mg) (1 M; filtrated on a 0.22 μm filter)

Milliq water (filtrated on a 0.22 μm filter)

3-(4,5-dimethylthiazol-2-yl)-2,5-diphenyltetrazolium bromide (MTT)

Sodium chloride solution 2 M (kept at 4 °C)

Sodium citrate (5% and 10% [w/v] in distilled water. Adjusted pH 7) (kept at 4 °C)

#### Equipment

Combitips

Cotton swab

Membrane filters, white polycarbonate, type HTTP, 0.2-μm pore size, 47-mm diameter

Hematocytometer, Neubauer chamber

Micropipettors, 10 −, 100-, and 1000-μL, with corresponding tips

Microplate reader

Light microscope, phase contrast

Microplates (96-well) (Corning 3361)

Repeater pipettes

Insulin syringe 26 G 1/2S (1 mL)

Microtube centrifuges (1.5 mL)

## Assay procedures

### Shrimp and hemolymph collection

1 Load the 1 mL syringes with 100 μL of anticoagulant 10% sodium citrate (w/v). Work at room temperature (25 °C).
2 Withdraw hemolymph from the ventral sinus of shrimp, which is located at the basis of the first abdominal segment. To avoid contamination, clean the area with a cotton swab soaked in ethanol 70% (v/v).
3 Mix hemolymph samples and adjust the dilution in anticoagulant to a final ratio of 50/50. Hemolymph samples are kept at room temperature until use, under aseptic conditions.

### Hemograma

1 Fix an aliquot of the hemolymph using formaldehyde 3.7%, in a V/V ratio.
2 Count the hemocytes using 10 μL of the fixed hemolymph with a hematocytometer (Neubauer chamber). Adjust hemocytes concentration to 1 × 10^7^ cells ml^−1^, using sodium citrate at 5% in sodium chloride solution 2%.

### Primary cell culture of shrimp hemocytes

1 Deposit the mixture of hemolymph- anticoagulant in 50 μL final volume in microplate wells. Activate the hemocytes with 50 μL/well of modified Hank's balanced salt solution (HBSS), containing HBSS 1x, 2.6 g l^−1^ HEPES, 85 mM NaCl, 12 mM Ca^2+^ and 26 mM Mg^2+^, pH 7.2 (MHBSS-3), filtrated on a 0.22 μm filter. Incubate for 60 min at room temperature for adherence of the hemocytes. Prepare fresh MHBSS, 30 min before use.
2 Eliminate the supernatants and immediately deposit 50 μL/well of MHBSS-2 containing HBSS 1X, 6 mM Ca^2+^ and 12 mM Mg^2+^ (pH 7.2).

### Exposure of hemocytes to assessed product

**Fig. 1:**
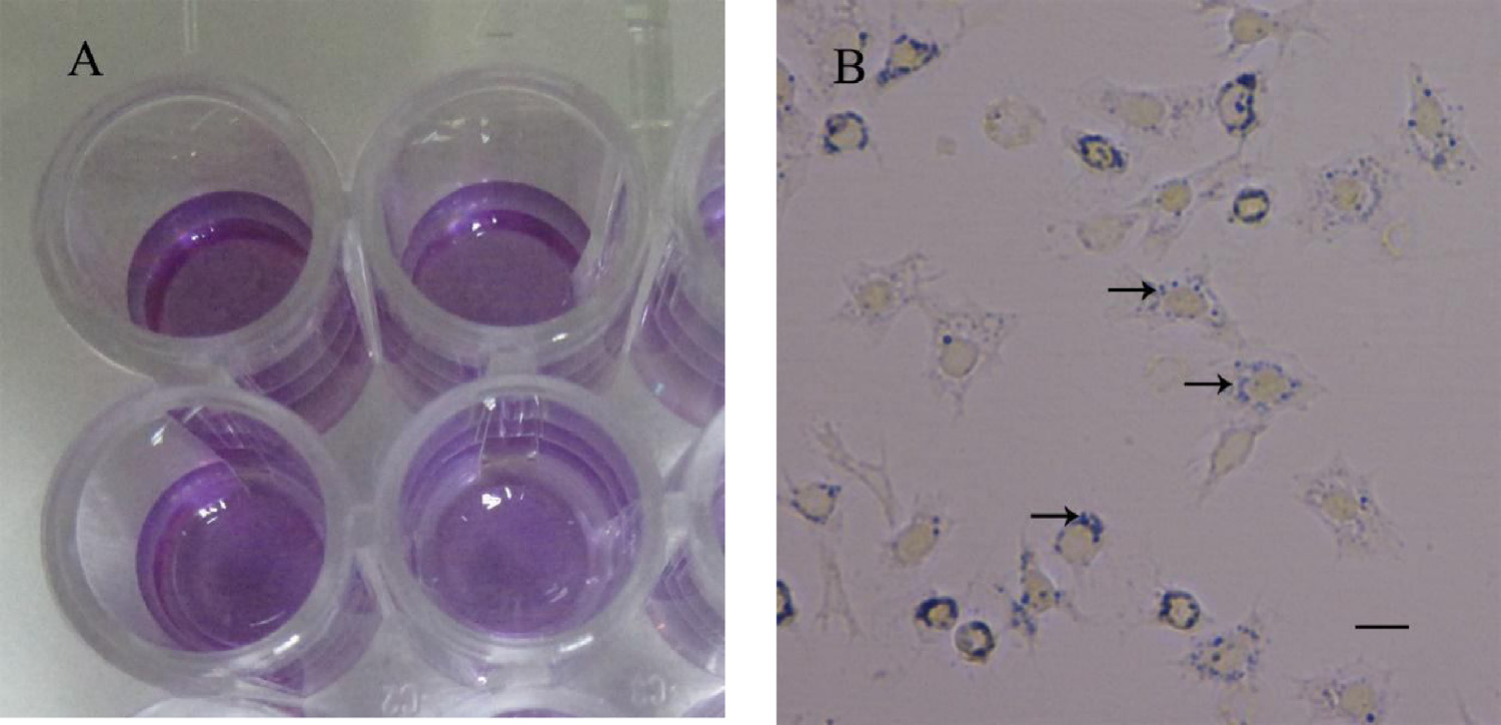
MTT reduction assay performed in shrimp haemocytes. (*A*), wells containing formazan crystals dissolved in isopropanol. (*B*), shrimp hemocytes stained with formazan (arrows). Scale bar, 100 μm.
4 Read at 620 nm in a microplate reader. The percentage of cell viability is obtained using the formula.

1 The product to be evaluated is dissolved in MHBSS-2 at different concentrations. Distribute 50 μL per well (minimum three replicates by dilution factor) onto hemocyte primary cultures. As a positive control (untreated cells) deposit by triplicate only 50 μL of MHBSS-2. Incubate for 90 min at 25 °C.
2 After incubation, add 10 μL of MTT (5 mg/ml MTT in milliq water) to all wells and incubate for 120 min at 25 °C. Keep in the dark.
3 After 120 min of incubation, shrimp hemocytes become stained with formazan and the wells turn purple (Fig. 1B). Remove the supernatant, and add 150 μL of isopropanol containing 0.04 N HCl. Homogenize vigorously to dissolve the formazan crystals (Fig. 1A), placing the microplate onto an ice bed.

**Cell viability OD** = (OD exposed cells/OD control cells) × 100%

**OD**: Optical density our 620 nm.

## Additional information

The toxicity assay developed by Mossman [2] has been applied in several studies with eukaryotic cells and it is widely used to measure cytotoxic, and antiproliferative activity of compounds [3]. This test has also been used by Jose et al. [4] to estimate the viability of hemocytes of *Penaeus monodon* shrimp to study the White Spot Syndrome Virus *in vitro*. In our study we used the procedure described by Muñoz et al. [5] to perform primary cell cultures of *P. vannamei* haemocytes. This method requires only a solution of MHBSS containing Ca^2+^ and Mg^2+^ to keep hemocytes metabolically active (Fig. 1B) to successfully carry out the cytotoxicity assay.

**Fig. 2:**
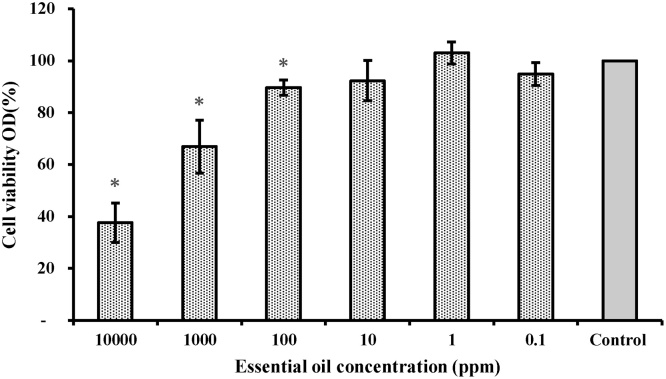
Toxicity assay performed with essential oil of *O. vulgare* over shrimp hemocytes (after 4 h). Viability of hemocytes in the control treatment was established at 100% and the other treatments were normalized accordingly. *,significantly different from control (P > 0.05, Duncan’s new multiple range test).

We used this assay to evaluate the toxic doses of different aquaculture additives, such as essential oils and antibiotics amongst others. In the (Fig. 2), we illustrated the effect of essential oil of *Origanum vulgare* (18% oil of *O. vulgare*), over shrimp hemocyte viability. *O. vulgare* essential oil is rich in thymol and carvacrol, phenolic compounds with several bioactivities [6], antioxidant [[7], [8], [9]], microbicidal [[10], [11], [12]] and immune modulator [13]. The results obtained with our *in vitro* test indicated that *O. vulgare* essential oil is not toxic for hemocyte at concentration 0.1, 1 and 10 ppm as no significant differences in cell viability were found between control and treatments. At these concentrations less than 10% of the hemocytes were affected (Fig. 2). Based on these results we performed an *in vivo* study. Post-larvae from *P. vannamei* shrimp in PL-1, were exposed to *O. vulgare* essential oil, using several doses between 0.1 and 10 ppm. The post-larvae survival was slightly affected 10% only at 10 ppm. These results were consistent with *in vitro* results, indicating the effectiveness of *in vitro* assay to determine no toxic therapeutic doses of functional additives as a pre-application process in *in vivo* trials. Also, we evaluated the toxicity *in vitro* of oxitetracicline (OTC) and florfenicol, antibiotics commonly used in aquaculture. Recording toxicity for oxytetracycline and florfenicol in shrimp hemocytes at concentrations of 4000 and 2000 μg/ml respectively. At these concentrations 15% of the cell viability was affected. Combining this information together with data of bacterial resistance, researchers and producers could effective doses of these drugs at commercial scale.

In conclusion, this test is a simple and reliable tool that requires few physical and economic resources to evaluate in short time (6 h) the cytotoxic effect of therapeutic products and additive to be included in shrimp culture.
